# Development and validation of an interpretable machine learning model for early prediction of postoperative atrial fibrillation following on-pump cardiac surgery

**DOI:** 10.3389/fsurg.2026.1933263

**Published:** 2026-09-10

**Authors:** Miaomiao Qian, Jie Yang, Dandan Geng

**Affiliations:** Department of Cardiovascular Surgery, The First Affiliated Hospital of Nanjing Medical University, Nanjing, China

**Keywords:** cardiac surgery, cholinesterase, machine learning, postoperative atrial fibrillation, random forest, shap

## Abstract

**Background:**

Postoperative atrial fibrillation (POAF) complicates 20%–40% of cardiac surgeries, increasing morbidity, length of stay, and healthcare costs. Early risk stratification could enable targeted prophylactic interventions. This study aimed to develop and validate an interpretable machine learning model for predicting incident POAF following on-pump cardiac surgery using routinely available perioperative parameters.

**Methods:**

We conducted a retrospective cohort study of 1,054 adults undergoing on-pump cardiac surgery between January and December 2025. The development cohort (*n* = 870) was randomly partitioned into training (70%) and internal validation (30%) sets, with a temporal validation cohort (*n* = 184) recruited from a later period at the same institution. Least absolute shrinkage and selection operator (LASSO) regression identified optimal predictors from 56 candidate variables. Six machine learning algorithms were evaluated, with random forest selected based on discriminative performance. Model interpretability was assessed using SHapley Additive exPlanations (SHAP) analysis.

**Results:**

POAF incidence was 27.1%. LASSO regression identified 11 optimal predictors, including operation duration, age, oxygenation indices, lactate, and cholinesterase (ChE). The random forest model achieved area under the curve values of 0.725 (training), 0.711 (internal validation), and 0.740 (temporal validation), indicating acceptable discrimination with stable performance across cohorts. SHAP analysis revealed ChE and age as the predominant risk drivers, with exploratory, model-derived nonlinear thresholds identified: ChE inflection at approximately 4,000 U/L, accelerated age risk beyond 70 years, and J-shaped lactate risk escalation above 5 mmol/L.

**Conclusions:**

This study presents an interpretable machine learning model for POAF prediction developed and temporally validated in a single-center cohort. The identification of ChE as a key predictive biomarker is hypothesis-generating and warrants further mechanistic and prospective investigation. The model may serve as a candidate decision-support tool for early ICU risk stratification, pending prospective multicenter validation of its generalizability and clinical utility.

## Introduction

Postoperative atrial fibrillation (POAF) represents the most frequently encountered arrhythmia following cardiac surgery, with reported incidence rates ranging from 20% to 40% among patients undergoing coronary artery bypass grafting (CABG) and valvular procedures (1). This complication substantially increases the risk of thromboembolic events, prolongs intensive care unit (ICU) and hospital length of stay, elevates healthcare costs, and is associated with increased short- and long-term mortality (2, 3). Despite advances in perioperative care and the recognition of multiple risk factors, the precise pathophysiological mechanisms underlying POAF remain incompletely elucidated, and effective preventive strategies remain limited (4).

The pathogenesis of POAF is multifactorial, involving complex interactions between patient-specific predisposing factors, intraoperative insults, and postoperative physiological disturbances (5). Established preoperative risk factors include advanced age, male sex, history of hypertension, left atrial enlargement, and reduced left ventricular ejection fraction (6). Intraoperative factors such as prolonged cardiopulmonary bypass time, aortic cross-clamping, and surgical manipulation of atrial tissue contribute to atrial inflammation and electrical remodeling (7). Postoperatively, systemic inflammatory responses, oxidative stress, autonomic dysregulation, and electrolyte imbalances create a proarrhythmic substrate that predisposes to atrial fibrillation development (8, 9).

Traditional risk stratification tools for POAF, including the HATCH score, CHA₂DS₂-VASc score, and various institution-specific algorithms, have demonstrated modest predictive performance and limited clinical utility (10, 11). These conventional models rely primarily on preoperative clinical characteristics and often fail to capture the dynamic physiological changes occurring during the perioperative period. Furthermore, the complex, nonlinear interactions between multiple risk factors challenge traditional statistical approaches that assume linear relationships and independence between variables.

Machine learning (ML) techniques offer significant advantages over conventional statistical methods for clinical prediction modeling by their ability to handle high-dimensional data, capture nonlinear relationships, and automatically learn complex feature interactions from training data (12, 13). Recent applications of ML in cardiovascular medicine have demonstrated promising results for risk prediction, outcome stratification, and clinical decision support (14).

Despite these advances, several critical gaps remain in the literature. First, many existing ML models for POAF prediction rely on preoperative variables alone, potentially missing important physiological information available in the immediate postoperative period (15). Second, the “black box” nature of many ML algorithms limits their clinical interpretability and adoption. Third, validation of proposed models beyond the development setting is often lacking, raising concerns about generalizability across different patient populations and healthcare settings. Finally, the identification of novel biomarkers with predictive value for POAF could provide insights into underlying pathophysiological mechanisms and potential therapeutic targets.

The present study addresses these limitations by developing and validating a ML-based predictive model for incident POAF using routinely available perioperative data, including immediate postoperative laboratory parameters. We employed least absolute shrinkage and selection operator (LASSO) regression for feature selection, compared multiple ML algorithms to identify the optimal approach, and utilized SHapley Additive exPlanations (SHAP) analysis to provide interpretable insights into model predictions. The model was internally validated and further assessed using temporal validation to evaluate the stability of its performance over time. We hypothesized that incorporating immediate postoperative physiological parameters would enhance predictive performance beyond traditional preoperative risk factors alone, and that ML interpretability methods would generate clinically testable hypotheses regarding POAF pathophysiology.

## Methods

### Study design and population

**Figure 1:**
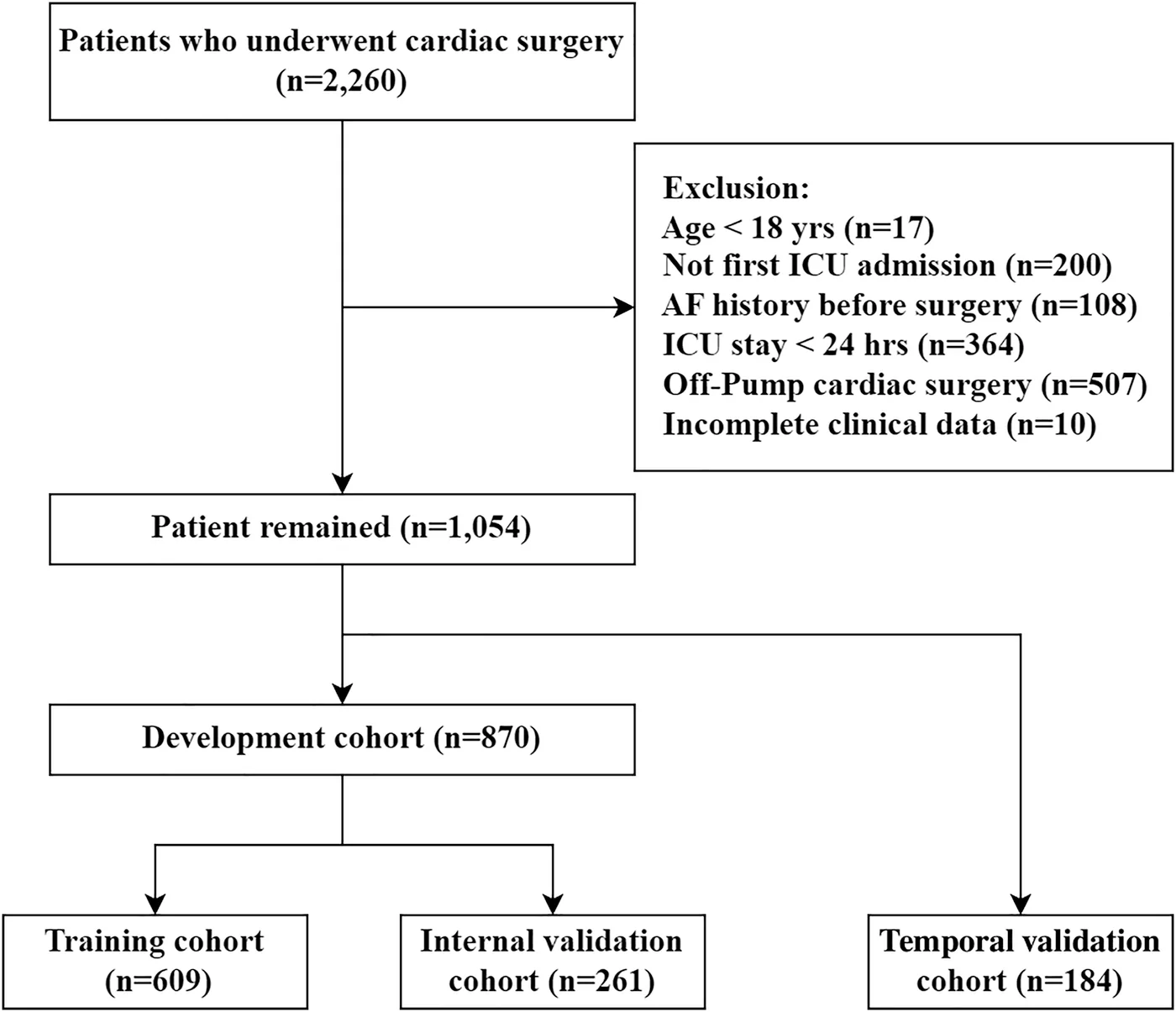
Study flow diagram. Of 2,260 patients undergoing cardiac surgery, 1,054 met eligibility criteria after exclusions for age <18 years (*n* = 17), non-first ICU admission (*n* = 200), preoperative AF (*n* = 108), ICU stay <24 h (*n* = 364), off-pump surgery (*n* = 507), and incomplete data (*n* = 10). The development cohort (*n* = 870) was partitioned into training (*n* = 609) and internal validation (*n* = 261) sets, with an independent temporal validation cohort (*n* = 184).

This retrospective cohort study was conducted at the Department of Cardiovascular Surgery, First Affiliated Hospital of Nanjing Medical University. Consecutive adult patients (≥18 years) who underwent on-pump cardiac surgery between January and December 2025 were screened. Inclusion criteria required postoperative ICU admission for ≥24 h as first-time admissions without a preoperative history of atrial fibrillation (AF). Exclusion criteria encompassed off-pump procedures, documented prior AF, non-first ICU admissions, ICU stay <24 h, incomplete perioperative data, or age <18 years. The ≥24 h ICU stay requirement was prespecified to guarantee a uniform window for predictor collection and a minimum period of continuous ECG monitoring for outcome ascertainment; we acknowledge that this criterion may preferentially exclude lower-risk patients with rapid, uncomplicated recovery, and this potential selection bias is addressed in the Limitations. Of 2,260 screened patients, 1,054 met eligibility (Figure 1). The development cohort (January–September 2025, *n* = 870) was randomly partitioned into a training set (70%, *n* = 609) for model derivation and hyperparameter tuning, and an internal validation set (30%, *n* = 261) for preliminary performance assessment (Supplementary Table S1). An independent temporal validation cohort (October–December 2025, *n* = 184) was reserved to evaluate the stability of model performance across a distinct temporal window (Supplementary Tables S2, S3). The study protocol was approved by the Institutional Review Board of the First Affiliated Hospital of Nanjing Medical University (approval number: 2025-SR-764). Given the retrospective nature of the analysis and the use of de-identified data extracted from electronic health records (EHR), the requirement for individual patient informed consent was waived. The study was conducted in accordance with the Declaration of Helsinki and reported following the Transparent Reporting of a multivariable prediction model for Individual Prognosis or Diagnosis (TRIPOD) guidelines.

### Outcome definition and ascertainment

The primary outcome was incident postoperative atrial fibrillation (POAF), defined as new-onset AF or atrial flutter lasting ≥30 s, documented on continuous telemetry or a 12-lead electrocardiogram (ECG) and confirmed by the treating physician. All patients underwent continuous ECG monitoring from ICU admission until ICU discharge, with daily review of rhythm records; transient episodes meeting the duration criterion were included. The observation period for outcome detection extended from ICU admission to ICU discharge. All predictor variables were obtained within the first hour after ICU admission, and all patients were in sinus rhythm at the time of predictor collection. Because POAF was defined as an incident event newly detected during subsequent continuous monitoring, all predictor measurements temporally preceded POAF onset in every patient, thereby excluding temporal leakage between predictors and outcome. POAF was coded as the positive class (event = 1) for all discrimination and precision-recall analyses. The same continuous monitoring and adjudication protocol was applied in the temporal validation cohort, and the time from ICU admission to the first POAF episode was recorded for every event.

### Data collection

Routinely available perioperative data were retrospectively extracted from institutional EHR. All laboratory measurements and physiological parameters were uniformly obtained within the first hour following ICU admission after on-pump cardiac surgery to ensure temporal consistency and minimize postoperative variation. Demographic variables included age, sex, height, weight, and body mass index (BMI), alongside total intraoperative duration. Immediately upon ICU arrival, vital signs comprising heart rate, respiratory rate, systolic/diastolic/mean arterial blood pressure (MAP), and peripheral oxygen saturation (SpO2) were recorded and extracted. Arterial blood gas (ABG) analysis within the 1 h window encompassed pH, partial pressure of carbon dioxide (PaCO2), partial pressure of oxygen (PaO2), arterial oxygen saturation (SaO2), base excess (BE), PaO2/FiO2 ratio, electrolytes (sodium, potassium, ionized calcium), and lactate (Lac) levels. Hematological assessments included complete blood count with full five-part differential (lymphocytes, monocytes, neutrophils, eosinophils, basophils), red cell indices (RBC, hemoglobin, hematocrit, mean corpuscular volume [MCV], mean corpuscular hemoglobin [MCH], mean corpuscular hemoglobin concentration [MCHC], red cell distribution width [RDW]), and platelet parameters (PLT, PCT, MPV, PDW). Coagulation profiles comprised prothrombin time (PT), international normalized ratio (INR), activated partial thromboplastin time (APTT), fibrinogen (FIB), thrombin time (TT), and D-dimer concentrations. Serum biochemistry covered serum electrolytes, renal function markers (blood urea nitrogen [BUN], creatinine), glucose, hepatic enzymes (alanine aminotransferase [ALT], aspartate aminotransferase [AST], alkaline phosphatase [ALP], gamma-glutamyl transferase [GGT], cholinesterase [ChE]), bilirubin fractions (total, direct, indirect), uric acid, and protein fractions (albumin, globulin, total protein). All laboratory analyses were performed using standardized automated analyzer platforms with rigorous internal and external quality control protocol. Serum cholinesterase was measured as butyrylcholinesterase (pseudocholinesterase) activity on the hospital's automated chemistry analyzer as part of the routine postoperative liver function panel (institutional reference range 4,650–10,440 U/L).

### Data preprocessing

All included patients had complete data for the 56 candidate variables; patients with incomplete perioperative data (*n* = 10) had been excluded during screening (Figure 1), and no imputation was therefore required. Continuous variables were standardized (z-score transformation) using training-cohort means and standard deviations, and categorical variables were one-hot encoded. Extreme values were verified against source records and retained when physiologically plausible. No resampling or class-balancing procedures were applied; class imbalance was addressed analytically by evaluating precision-recall metrics, with POAF as the positive class, in addition to ROC-based metrics.

### Feature selection and machine learning model development

Feature selection was performed using LASSO logistic regression implemented in R (version 4.5.2) with the glmnet package. Twenty-fold cross-validation was employed to determine the optimal regularization parameter (*λ*) that minimized binomial deviance, and the one-standard-error rule (*λ*.1se) was applied to obtain a parsimonious model. The *λ*.1se rule was preferred over *λ*.min because it yields a sparser predictor set at the cost of only a minimal increase in cross-validated deviance, thereby reducing overfitting risk and improving clinical usability given the moderate number of outcome events. This approach shrinks less informative variable coefficients toward zero, thereby enabling automated selection of the most predictive features while mitigating overfitting and multicollinearity among the candidate predictors. The ML part of this study was conducted using the R package “mlr3.” Six predictive models were developed using the following algorithms: logistic regression (LR), decision tree (DT), support vector machine (SVM), extreme gradient boosting (XGBoost), random forest (RF), and naive Bayes (NB). Hyperparameter optimization was performed within the mlr3 ecosystem using the auto_tuner framework (mlr3tuning) with random search (batch size 10) terminated after 50 evaluations, using five-fold cross-validation with classification accuracy as the optimization metric and fixed random seeds for all stochastic procedures. All feature selection, preprocessing parameter estimation, and hyperparameter tuning were performed exclusively within the training cohort. The six candidate algorithms were compared in the internal validation set, at which stage random forest was selected; the temporal validation cohort was not accessed for any modelling decision and was reserved for a single, final evaluation. The final random forest model was refitted on the entire development cohort using the optimized hyperparameters before temporal validation. The models were evaluated using the area under the curve (AUC) of the receiver operating characteristic (ROC) curves to determine their predictive performance. Precision-recall analyses were conducted with POAF coded as the positive class (event = 1); the area under the precision-recall curve (PRAUC) was computed as average precision (AP), i.e., the weighted mean of precision across recall thresholds, rather than by trapezoidal interpolation. No-skill reference lines correspond to the event prevalence of each cohort (25.8%, 30.3%, and 21.2% for the training, internal validation, and temporal validation cohorts, respectively). The 95% confidence intervals for ROC-AUC and PRAUC were estimated with 1,000 bootstrap resamples. Model calibration was assessed in the internal and temporal validation cohorts using calibration curves with locally weighted (LOESS) smoothing and decile-based mean observed frequencies, the calibration intercept (target 0) and calibration slope (target 1) derived from logistic recalibration of the predicted log-odds, and the Brier score. Hyperparameter tuning was performed over the following random forest search space: number of trees (200–1,000), mtry (1–10), min.node.size (1–20), max.depth (5–30), sample.fraction (0.5–1.0), sampling with or without replacement (replace), and split rule (gini or extratrees), with a fixed evaluation budget of 50 evaluations. Analyses were conducted in R version 4.5.2 using mlr3 (1.3.0), mlr3tuning (1.5.1), glmnet (4.1.10), and ranger (0.18.0). LASSO feature selection was performed once on the complete training cohort before model tuning, and the selected features were held fixed during hyperparameter tuning; we acknowledge that this approach does not propagate feature-selection uncertainty into the reported performance estimates, which is addressed in the Limitations.

### Model interpretation and SHAP analysis

SHAP analysis was used to interpret the ML models and assess the contribution of each feature. The SHAP values for each feature were computed to provide an interpretable understanding of the model's decision-making process at both cohort and individual levels. Features were ranked based on their SHAP values, and SHAP summary and dependency plots were generated to visually demonstrate the relationship between the predictive features and the outcome. SHAP values describe how the fitted model uses predictors and were interpreted as exploratory, model-derived patterns; they were not used to infer causality, independent risk factors, biological mechanisms, or validated clinical cut-off values.

## Results

### Baseline characteristics of the study population

Between January and December 2025, 2,260 patients underwent cardiac surgery, of whom 1,054 met eligibility criteria. The development cohort and temporal validation cohort were partitioned as previously described (Figure 1). Within the development cohort, 236 patients (27.1%) developed incident AF during the index ICU stay. POAF occurred in 157 patients (25.8%) in the training cohort, 79 (30.3%) in the internal validation cohort, and 39 (21.2%) in the temporal validation cohort (Supplementary Tables S1–S3). Patients who developed AF were significantly older (64.7 ± 10.7 vs. 60.5 ± 11.4 years, *p* < 0.001) and underwent longer operations (320.0 ± 100.1 vs. 285.2 ± 94.7 min, *p* < 0.001) compared to those without AF. Additionally, SpO2 was significantly lower in the AF group (98.6 ± 2.3% vs. 99.3 ± 1.5%, *p* < 0.001). No significant between-group differences were observed in sex distribution, BMI, operation type, or immediate postoperative hemodynamic parameters including heart rate, respiratory rate, or MAP (Table 1). In the development cohort, the median time from ICU admission to the first POAF episode was 71 h (interquartile range 56–85 h); in the temporal validation cohort, the median time was 69 h (interquartile range 53–81 h).

**Table 1:** General characteristics and vital signs of patients in development cohort.

Variable	Total (*n* = 870)	Non-POAF (*n* = 634)	POAF (*n* = 236)	*p*-Value
Age (yr)	61.62 (11.38)	60.47 (11.42)	64.72 (10.72)	**<0**.001*
Gender (%)				1.000
Female	364 (41.8)	265 (41.8)	99 (41.9)	
Male	506 (58.2)	369 (58.2)	137 (58.1)	
Height (m)	1.65 (0.08)	1.65 (0.08)	1.65 (0.08)	0.645
Weight (kg)	66.40 (12.21)	66.60 (12.24)	65.89 (12.13)	0.448
BMI (kg/m^2^)	24.29 (3.58)	24.33 (3.60)	24.16 (3.54)	0.522
Operation type (%)				0.090
CABG	46 (5.3)	36 (5.7)	10 (4.2)	
Valve surgery	538 (61.8)	384 (60.6)	154 (65.3)	
Cardiac tumor resection	29 (3.3)	25 (3.9)	4 (1.7)	
LVAD implantation	16 (1.8)	8 (1.3)	8 (3.4)	
Atrial or ventricular septal defect repair	29 (3.3)	25 (3.9)	4 (1.7)	
Total aortic arch replacement	6 (0.7)	4 (0.6)	2 (0.8)	
Combined procedures	206 (23.7)	152 (24.0)	54 (22.9)	
Operation Duration (min)	294.59 (97.33)	285.15 (94.65)	319.97 (100.06)	**<0**.001*
HR (bpm)	93.24 (15.64)	93.01 (15.04)	93.85 (17.18)	0.482
RR	14.84 (2.12)	14.82 (2.03)	14.89 (2.37)	0.656
SBP (mmHg)	122.32 (19.08)	122.85 (19.21)	120.88 (18.69)	0.176
DBP (mmHg)	65.53 (11.43)	65.69 (11.48)	65.09 (11.30)	0.494
MBP (mmHg)	84.46 (12.55)	84.74 (12.71)	83.69 (12.09)	0.270
SpO2 (%)	99.09 (1.76)	99.26 (1.47)	98.64 (2.32)	**<0**.001*

Data are expressed as mean (SD) for continuous variables and percentage (%) for categorical variables. *P* values were calculated using Student's t-test for continuous variables and Pearson's chi-square test for categorical variables. Bold values indicate statistical significance (*P* < 0.05).

* *P* < 0.05.

BMI, Body Mass Index; CABG, coronary artery bypass grafting; LVAD, left ventricular assist device; HR, Heart Rate; RR, Respiratory Rate; SBP, Systolic Blood Pressure; DBP, Diastolic Blood Pressure; MBP, Mean Blood Pressure.

### Postoperative laboratory profiles

Laboratory analyses performed within one hour of ICU admission revealed multiple significant differences between AF and non-AF patients, which are summarized by physiological function in Table 2. Regarding metabolic and perfusion markers, Lac levels were significantly elevated in AF patients (4.37 ± 3.65 vs. 3.55 ± 2.38 mmol/L, *p* < 0.001). Hepatic function markers also showed notable differences, specifically lower ChE levels in the AF group (5,151.8 ± 928.5 vs. 5,533.7 ± 900.1 U/L, *p* < 0.001), alongside differences in bilirubin fractions and GGT. Respiratory and oxygenation parameters, including pH, PaO2, SaO2, and the PaO2/FiO2 ratio, were significantly lower in AF patients (*p* < 0.05). Hematological and coagulation assessments revealed differences in monocyte counts, red cell indices (MCV, RDW), platelet parameters, and D-dimer concentrations (*p* < 0.05).

**Table 2:** Results of ABG, blood counts, coagulation function test and serum biochemical parameters of patients in development cohort.

Variable	Total (*n* = 870)	Non-POAF (*n* = 634)	POAF (*n* = 236)	*p*-Value
pH	7.41 (0.08)	7.41 (0.08)	7.40 (0.08)	**0**.017*
PaCO2 (mmHg)	37.85 (7.46)	37.58 (7.31)	38.56 (7.83)	0.086
PaO2 (mmHg)	170.56 (75.99)	174.96 (77.26)	158.72 (71.28)	**0**.005*
SaO2 (%)	98.70 (2.38)	98.87 (1.85)	98.25 (3.37)	**0**.001*
BE (mmol)	−0.69 (3.29)	−0.60 (3.18)	−0.92 (3.55)	0.198
PaO2/FiO2	298.83 (131.64)	306.86 (130.85)	277.24 (131.58)	**0**.003*
ABG-Na (mmol)	140.21 (5.56)	140.05 (4.05)	140.62 (8.36)	0.177
ABG-K (mmol)	4.10 (0.54)	4.08 (0.54)	4.14 (0.55)	0.185
ABG-Ca (mmol)	1.13 (0.07)	1.13 (0.07)	1.13 (0.07)	0.826
Lac (mmol)	3.77 (2.80)	3.55 (2.38)	4.37 (3.65)	**<0**.001*
WBC (10^9^/L)	12.00 (4.41)	11.96 (4.26)	12.11 (4.80)	0.666
Lymphocyte (10^9^/L)	0.86 (0.57)	0.86 (0.58)	0.84 (0.57)	0.615
Monocyte (10^9^/L)	0.49 (0.34)	0.46 (0.32)	0.55 (0.40)	**0**.001*
Neutrophil (10^9^/L)	10.62 (3.99)	10.60 (3.88)	10.68 (4.29)	0.795
Eosinophil (10^9^/L)	0.03 (0.04)	0.03 (0.03)	0.03 (0.05)	0.763
Basophil (10^9^/L)	0.01 (0.01)	0.01 (0.01)	0.01 (0.01)	0.782
RBC (10^12^/L)	3.52 (0.48)	3.53 (0.48)	3.47 (0.47)	0.100
Hb (g/L)	106.01 (14.67)	106.35 (14.87)	105.09 (14.11)	0.263
HCT (%)	32.47 (4.36)	32.52 (4.45)	32.34 (4.14)	0.574
MCV (fL)	92.52 (5.00)	92.21 (4.89)	93.35 (5.21)	**0**.003*
MCH (pg)	30.19 (1.80)	30.14 (1.76)	30.34 (1.91)	0.155
MCHC (g/L)	326.35 (8.87)	326.92 (8.53)	324.84 (9.58)	**0**.002*
RDW (%)	13.26 (1.49)	13.16 (1.43)	13.51 (1.64)	**0**.002*
PLT (10^9^/L)	135.31 (45.56)	137.72 (43.37)	128.83 (50.53)	**0**.010*
PCT (%)	0.14 (0.04)	0.14 (0.04)	0.13 (0.05)	**0**.011*
MPV (fL)	10.30 (1.12)	10.28 (1.15)	10.33 (1.04)	0.594
PDW (%)	16.07 (1.18)	16.08 (1.18)	16.07 (1.19)	0.945
PT (s)	13.43 (1.58)	13.43 (1.66)	13.44 (1.34)	0.966
INR	1.18 (0.15)	1.18 (0.16)	1.18 (0.13)	0.964
APTT (s)	28.69 (7.24)	28.62 (7.75)	28.88 (5.66)	0.644
FIB (g/L)	2.72 (0.80)	2.74 (0.81)	2.68 (0.77)	0.312
TT (s)	20.60 (14.35)	20.41 (14.05)	21.11 (15.15)	0.518
D-dimer(mg/L)	4.39 (5.83)	4.09 (5.53)	5.21 (6.51)	**0**.012*
K (mmol)	4.38 (0.57)	4.37 (0.56)	4.40 (0.58)	0.472
Na (mmol)	141.92 (4.07)	141.77 (4.18)	142.33 (3.72)	0.075
Cl (mmol)	105.42 (4.46)	105.41 (4.51)	105.46 (4.31)	0.895
Ca (mmol)	2.35 (0.15)	2.35 (0.15)	2.35 (0.16)	0.688
ALT (U/L)	39.40 (48.12)	38.47 (49.38)	41.90 (44.55)	0.349
AST (U/L)	92.65 (99.75)	89.96 (97.08)	99.87 (106.48)	0.193
BUN (mmol)	8.88 (3.37)	8.66 (3.36)	9.47 (3.33)	**0**.001*
Cr (umol)	86.77 (72.76)	85.36 (75.16)	90.55 (65.90)	0.350
GLU (mmol)	10.50 (2.90)	10.44 (2.77)	10.65 (3.24)	0.347
TBIL (umol)	33.48 (12.54)	33.06 (12.00)	34.61 (13.87)	0.105
DBIL (umol)	10.78 (6.87)	10.43 (6.40)	11.73 (7.95)	**0**.013*
IBIL (umol)	22.70 (9.36)	22.63 (9.18)	22.88 (9.83)	0.725
ALP (U/L)	52.20 (20.65)	52.47 (20.69)	51.49 (20.56)	0.535
GGT (U/L)	32.65 (32.00)	31.05 (31.25)	36.97 (33.62)	**0**.015*
ChE (U/L)	5,430.13 (923.09)	5,533.73 (900.06)	5,151.79 (928.49)	**<0**.001*
UA (umol)	320.30 (74.71)	320.36 (75.68)	320.15 (72.19)	0.971
TP (g/L)	64.41 (7.09)	64.70 (7.01)	63.63 (7.25)	**0**.046*
GLB (g/L)	23.01 (3.99)	23.02 (3.83)	22.96 (4.41)	0.841
ALB (g/L)	41.43 (5.14)	41.71 (5.00)	40.67 (5.43)	**0**.008*

Data are expressed as mean (SD) and *P* value was tested by Student's *t*-test. Bold values indicate statistical significance (*P* < 0.05).

* *P* < 0.05.

ABG, Arterial Blood Gas; BE, Base Excess; Lac, Lactate; WBC, White Blood Cell; RBC, Red Blood Cell; Hb, Hemoglobin; HCT, Hematocrit; MCV, Mean Corpuscular Volume; MCH, Mean Corpuscular Hemoglobin; MCHC, Mean Corpuscular Hemoglobin Concentration; RDW, Red Cell Distribution Width; PLT, platelet; PCT, Plateletcrit; MPV, Mean Platelet Volume; PDW, Platelet Distribution Width; PT, Prothrombin Time; INR, International Normalized Ratio; APTT, Activated Partial Thromboplastin Time; FIB, Fibrinogen; TT, Thrombin Time; ALT, Alanine Transaminase; AST, Aspartate Transaminase; BUN, Blood Urea Nitrogen; Cr, Creatinine; GLU, Glucose; TBIL, Total Bilirubin; DBIL, Direct Bilirubin; IBIL, Indirect Bilirubin; ALP, Alkaline Phosphatase; GGT, Gamma-Glutamyl Transferase; ChE, Cholinesterase; UA, Uric Acid; TP, Total Protein; GLB, Globulin; ALB, Albumin.

### Feature selection

**Figure 2:**
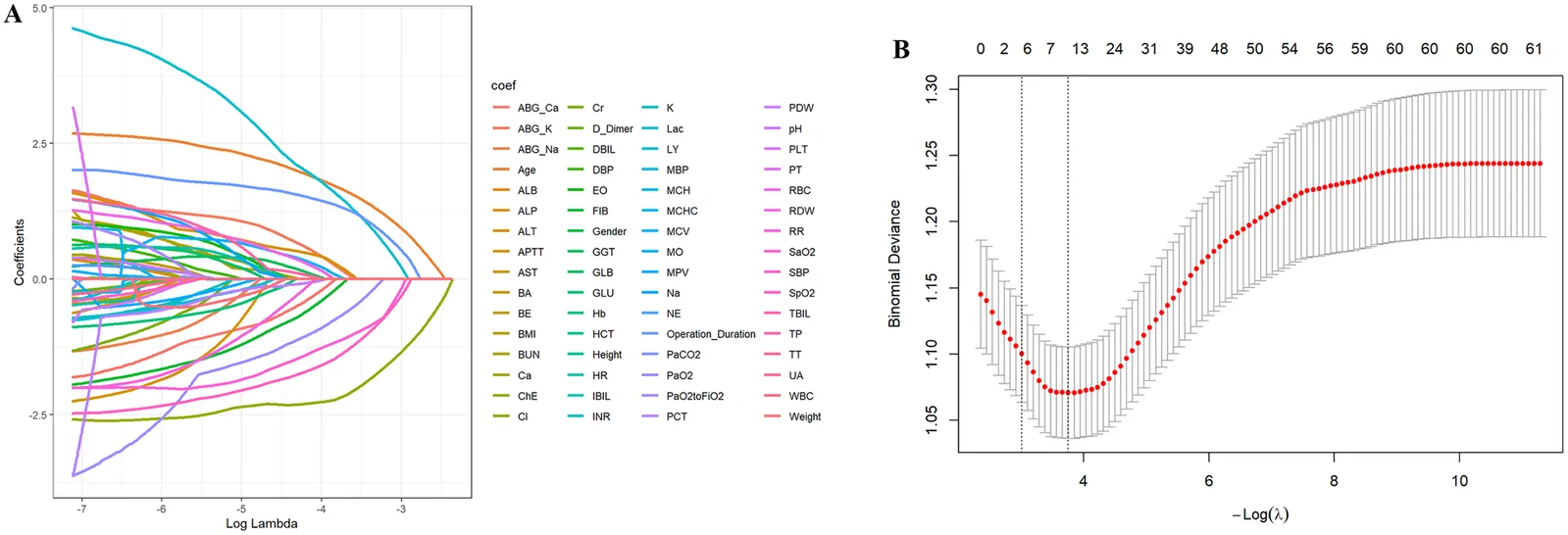
Feature selection using LASSO regression. **(A)** Coefficient paths of candidate variables across log(lambda) values. **(B)** Twenty-fold cross-validation for optimal lambda selection based on binomial deviance. Vertical dotted lines indicate lambda.min and lambda.1se. Application of the one-standard-error rule identified 11 variables with non-zero coefficients for subsequent model development.

Prior to multivariable model development, univariate predictive performance was assessed for each candidate variable (Supplementary Figures S1A, B). ChE, age, and operation duration demonstrated the highest discriminative ability (AUC >0.58), while SpO2 exhibited limited discriminative performance (AUC 0.42). To mitigate overfitting and enhance model parsimony, LASSO regression with 20-fold cross-validation was applied to the training cohort. The regularization parameter (*λ*) was optimized using the one-standard-error rule (*λ* = 0.018, log[*λ*] = −4.0) (Figures 2A,B). This approach yielded a sparse solution from 56 candidate variables, identifying 11 non-zero coefficients as optimal predictors: operation duration, age, SpO2, PaO2, SaO2, ABG K+, Lac, MCV, FIB, ALT, and ChE. These 11 variables were subsequently entered as candidate features for ML model development.

### Machine learning model selection

**Figure 3:**
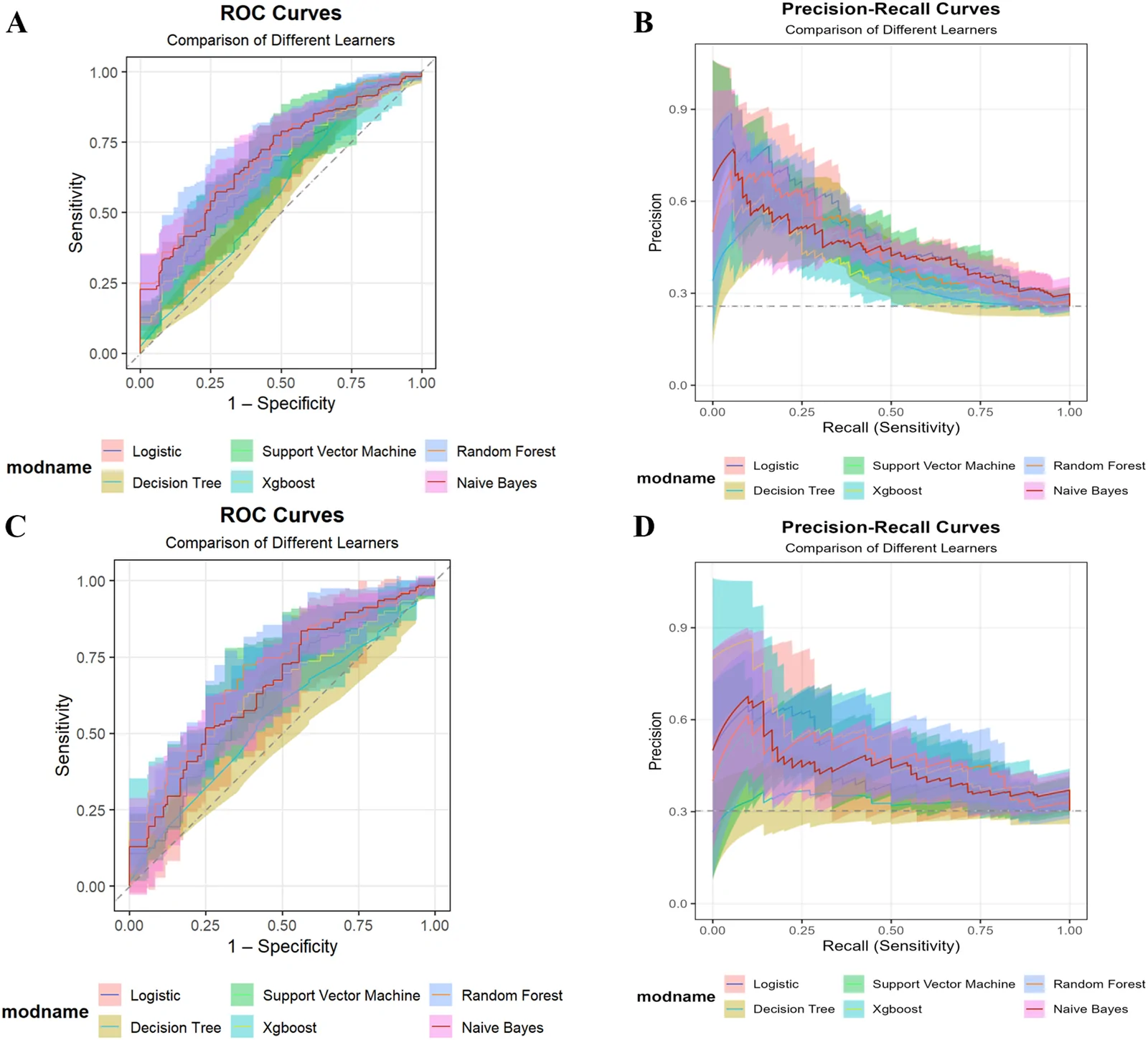
Discriminative performance of machine learning classifiers. **(A,B)** ROC and precision-recall curves in the training cohort (*n* = 609). **(C,D)** ROC and precision-recall curves in the internal validation cohort (*n* = 261). All classifiers were evaluated with default (untuned) hyperparameter settings at the model-development stage; the performance of the final tuned RF model is presented in Supplementary Figure S2. Precision-recall curves were generated with POAF coded as the positive class (event = 1); PRAUC was computed as average precision; dashed lines indicate the no-skill prevalence reference (0.258 for the training cohort and 0.303 for the internal validation cohort). Training-cohort performance was estimated using out-of-fold cross-validation predictions.

To identify the optimal algorithm, 6 ML classifiers—LR, DT, SVM, XGBoost, RF, and NB—were first trained and internally validated using the 11 LASSO-selected features with default (untuned) hyperparameter settings during the model-development stage. Model discrimination was assessed using AUC-ROC, while precision-recall area under the curve (PRAUC) was evaluated given the class imbalance (27.1% event rate). All precision-recall analyses were performed with POAF explicitly coded as the positive class, and no-skill reference lines corresponding to class prevalence were added to the plots. As depicted in Figure 3 and quantified in Table 3, the RF model exhibited the most optimal performance across all evaluation metrics, particularly maintaining a high AUC value in the temporal validation set, which indicates acceptable discrimination and comparatively stable performance across cohorts. In the training cohort (Figures 3A,B), RF achieved an AUC of 0.712 and PRAUC of 0.439 (95% CI 0.366–0.521, based on out-of-fold cross-validation predictions), outperforming LR (AUC 0.649), DT (AUC 0.566), and XGBoost (AUC 0.655). While SVM exhibited a comparable training AUC (0.651), RF maintained more consistent discrimination in the internal validation cohort (AUC 0.692) and achieved a PRAUC of 0.469 (95% CI 0.371–0.585) in the internal validation cohort, comparable to alternative algorithms such as NB (0.426) and SVM (0.398) (Figures 3C,D). Consequently, RF was selected as the final predictive model. It should be noted that the discriminative advantage of RF over LR was modest (training AUC 0.712 vs. 0.649), and the corresponding curves overlapped substantially in the internal validation cohort; RF was therefore selected primarily for the consistency of its performance across cohorts rather than for a definitive superiority over simpler approaches. Of note, all metrics reported in Figure 3 and Table 3 were obtained prior to hyperparameter tuning; the performance of the final optimized RF model is presented in the following section (Supplementary Figure S2).

**Table 3:** Evaluation of model performance in training and internal validation cohorts.

Model	Training Cohort				Internal Validation Cohort			
	Accuracy	AUC	Sensitivity	PRAUC	Accuracy	AUC	Sensitivity	PRAUC
Logistic	0.753	0.649	0.950	0.437	0.720	0.622	0.957	0.420
Decision Tree	0.724	0.566	0.893	0.375	0.644	0.554	0.808	0.365
Support Vector Machine	0.744	0.651	0.966	0.424	0.686	0.671	0.972	0.398
XGBoost	0.726	0.655	0.888	0.370	0.652	0.645	0.802	0.405
Random Forest	0.754	0.712	0.944	0.480	0.686	0.692	0.904	0.457
Naive Bayes	0.732	0.697	0.904	0.429	0.697	0.658	0.833	0.426

All models were evaluated with default (untuned) hyperparameter settings during the model-development stage; the performance of the final tuned RF model (AUC 0.725 in the training cohort and 0.711 in the internal validation cohort) is reported in Supplementary Figure S2 of the main text. PRAUC, precision-recall area under the curve (computed as average precision with POAF coded as the positive class); AUC, area under the receiver operating characteristic curve; CI, confidence interval.

### Hyperparameter optimization of random forest model

To enhance model generalizability and mitigate overfitting, automated hyperparameter tuning was performed using the mlr3 package with random search via the auto_tuner framework, as described in the Methods. Optimal hyperparameters for the RF model were identified as: 621 trees, mtry = 5, min.node.size = 13, max.depth = 19, and sample.fraction = 0.974. Following optimization, the RF model achieved an AUC of 0.725 in the training cohort (Supplementary Figure S2A). Internal validation performance remained stable with an AUC of 0.711 (Supplementary Figure S2B). The tight concordance between training and validation performance (*Δ*AUC = 0.014) suggests that overfitting was limited following optimization.

### SHAP model analysis

**Figure 4:**
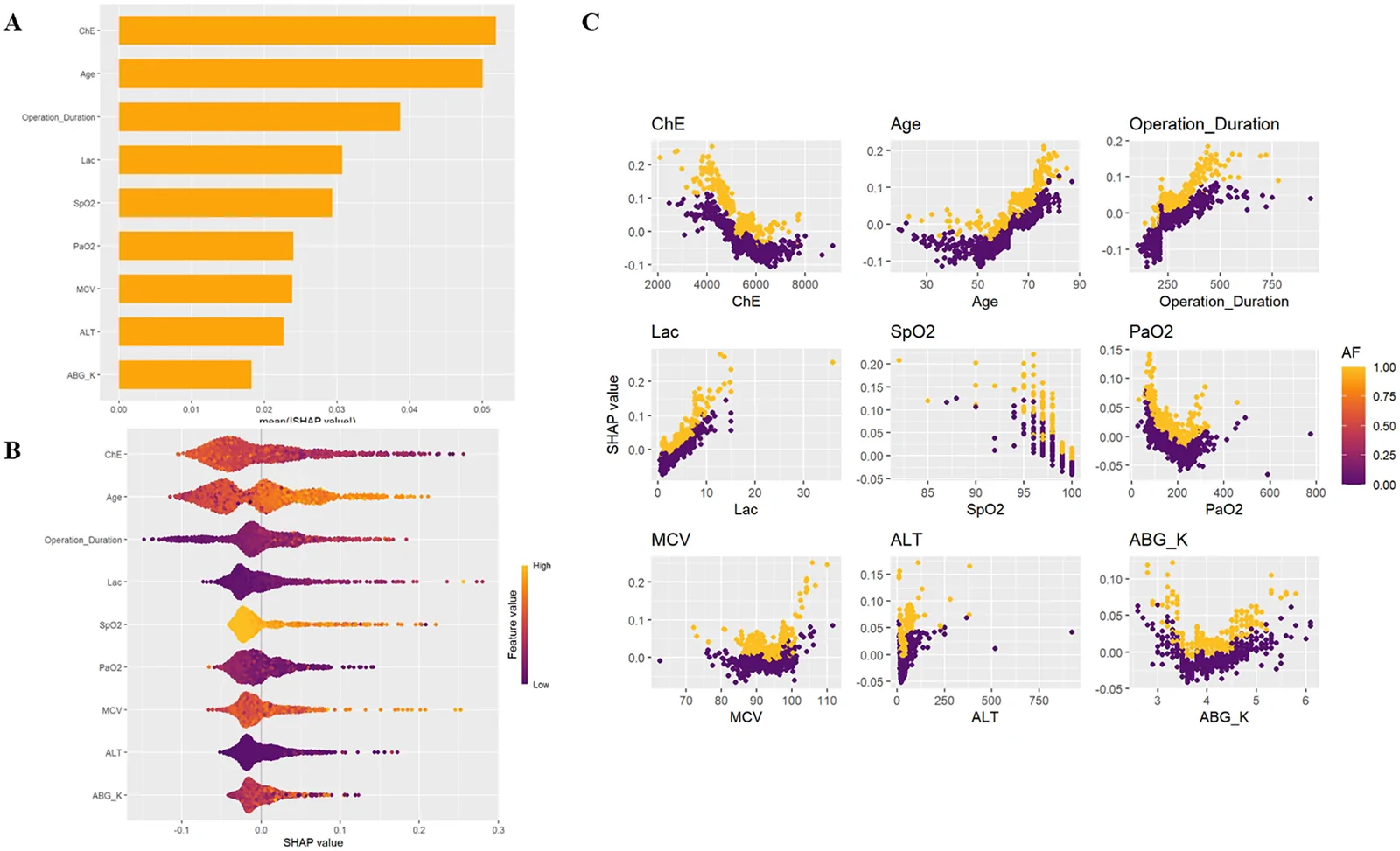
SHAP analysis of the random forest model. **(A)** Global feature importance ranked by mean absolute SHAP values. **(B)** SHAP summary plot showing directional effects of top features (color indicates feature value). **(C)** SHAP dependence plots revealing nonlinear relationships between key predictors and outcome risk; inflection points are exploratory, model-derived patterns without formal breakpoint estimation. The exploratory t-SNE visualization of patient-level SHAP vectors has been moved to the Supplementary Material (Supplementary Figure S3).

SHAP analysis was employed to delineate the hierarchical importance and directional effects of clinical predictors for postoperative AF. Global feature importance, quantified by mean absolute SHAP values, identified ChE and age as the predominant drivers of AF risk, followed by operation duration, Lac, and SpO2 (Figure 4A). Directional analysis revealed that ChE exhibited a robust inverse association with model-predicted AF risk, while age demonstrated a monotonic positive association. SpO2 showed a negative correlation, identifying low oxygen saturation as a contributing factor (Figure 4B).

Detailed examination of dependency structures suggested exploratory, nonlinear risk patterns (Figure 4C). ChE displayed an L-shaped curve with an inflection point at approximately 4,000 U/L, below which AF risk increased precipitously. Age exhibited an accelerated risk gradient beyond 70 years. Lac demonstrated a J-shaped dose-response, with risk escalating exponentially above 5 mmol/L. PaO2 exhibited an inverted U-shaped relationship, with 100–300 mmHg representing the lowest risk range. ABG K + showed a U-shaped pattern, with 3.5–4.5 mEq/L constituting the nadir of risk. MCV and ALT exhibited elevated risk confined to extreme values (>100 fL and >500 U/L, respectively). These inflection points were visually derived from SHAP dependency plots without formal breakpoint estimation or uncertainty assessment, and are therefore reported as exploratory, model-derived patterns rather than established clinical cut-off values.

As an exploratory visualization, patient-level SHAP vectors from the development cohort were embedded using t-distributed stochastic neighbor embedding (t-SNE; perplexity [5], random seed 2025) (Supplementary Figure S3). AF patients appeared to form a relatively concentrated region distinct from most non-AF individuals. This visualization is descriptive only and does not constitute independent validation of model discrimination; we note that the apparent visual separation exceeds what the corresponding discrimination metrics (AUC 0.71–0.74) would imply, and t-SNE distances and cluster boundaries should not be over-interpreted. Collectively, SHAP analysis identified ChE and age as the primary model-derived drivers of postoperative AF; the observed nonlinear patterns are exploratory and hypothesis-generating rather than validated clinical thresholds.

### Temporal validation and performance stability

**Figure 5:**
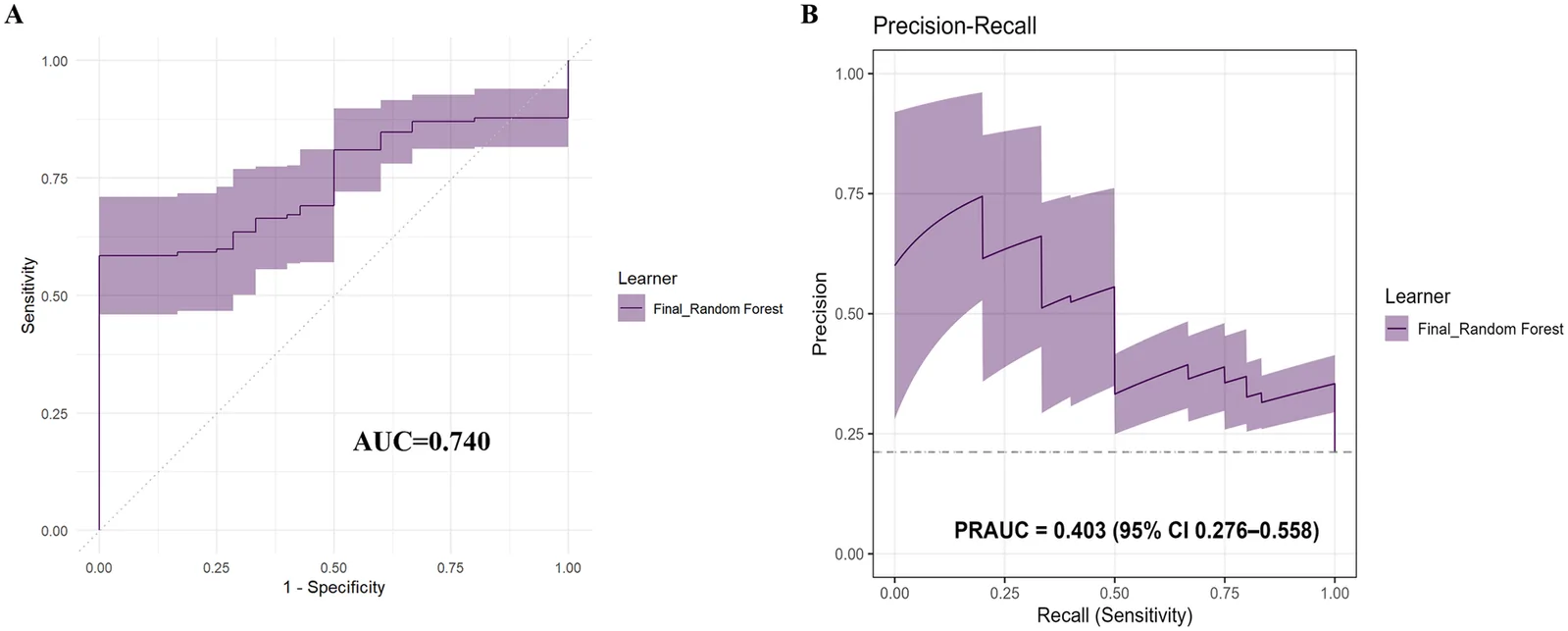
Temporal validation performance. **(A)** ROC curve (AUC = 0.740). **(B)** Precision-recall curve (PRAUC = 0.403 (95% CI 0.276–0.558), computed as average precision with POAF as the positive class; the dashed line indicates the no-skill prevalence reference (0.212)) in the temporal validation cohort (*n* = 184). Shaded areas represent 95% confidence intervals.

The final model was evaluated once in the temporal validation cohort, after all modelling decisions had been finalized. The optimized RF achieved an AUC of 0.740 (Figure 5A), closely matching internal validation and indicating stable discrimination across the temporal shift within our institution. Given the class imbalance, precision-recall performance, computed with POAF coded as the positive class, yielded a PRAUC of 0.403 (95% CI 0.276–0.558) (Figure 5B), with the no-skill prevalence reference line (0.212) shown for comparison. The minimal performance degradation between cohorts (*Δ*AUC = 0.029) suggests the stability of the model in identifying the AF class.

**Figure 6:**
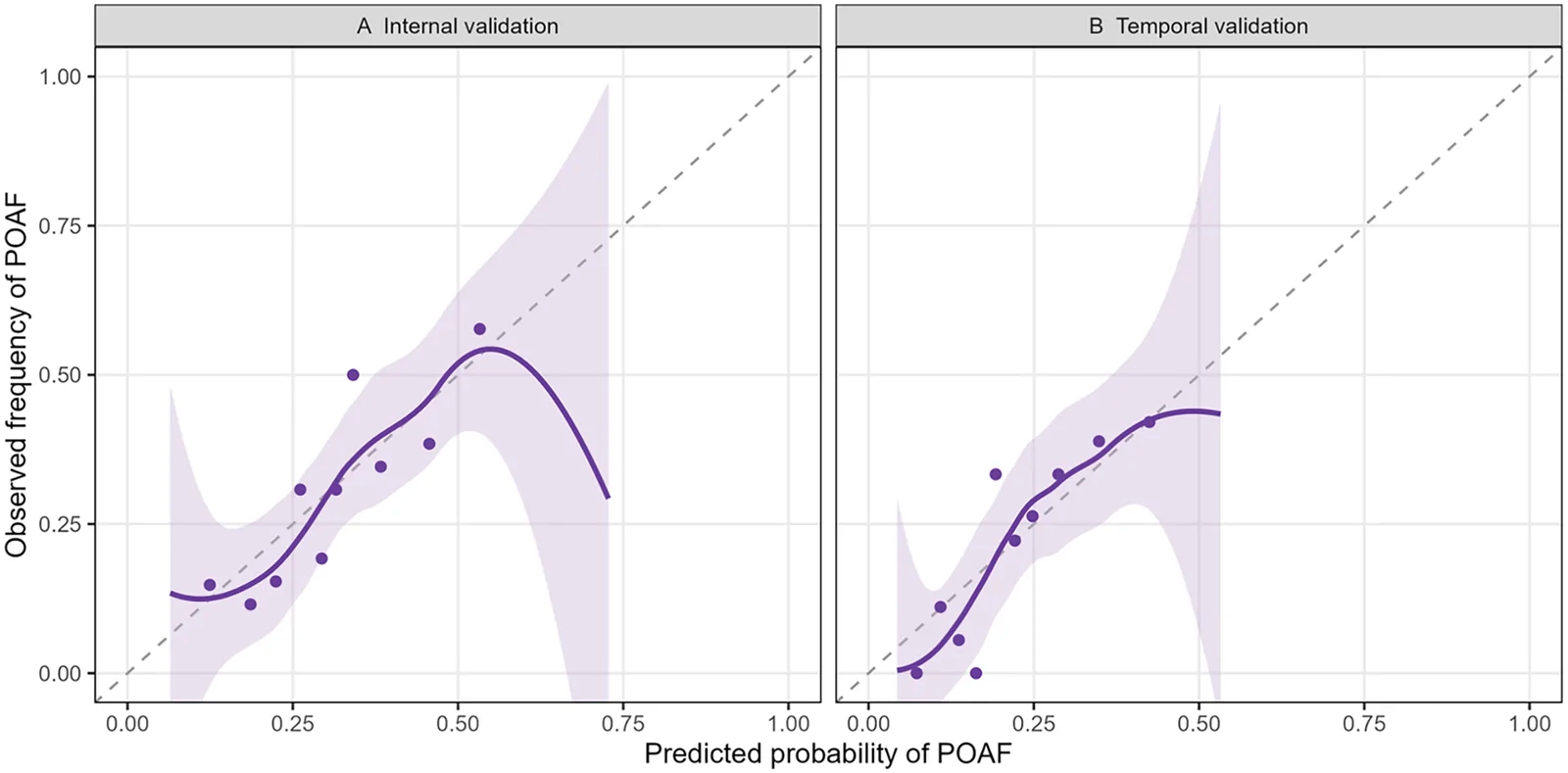
Calibration of the random forest model in the internal **(A)** and temporal **(B)** validation cohorts, with POAF coded as the positive class. LOESS-smoothed calibration curves (solid lines) and decile-based mean observed frequencies (dots) are shown against perfect calibration (dashed diagonal); the calibration intercept, calibration slope, and Brier score are reported in the Results.

Model calibration is presented in Figure 6. In the internal validation cohort, the calibration intercept was −0.043, the calibration slope was 1.077, and the Brier score was 0.195, indicating good calibration. In the temporal validation cohort, the calibration intercept was −0.054, the calibration slope was 1.400, and the Brier score was 0.150; the slope above 1 indicates that predicted risks were under-dispersed (insufficiently spread) in the temporal cohort, while calibration-in-the-large remained good in both cohorts.

## Discussion

The core finding of this study is that a ML model combining immediate postoperative laboratory indicators predicts POAF with moderate accuracy, with ChE and age serving as key drivers. The incidence of POAF in our cohort (27.1%) aligns with contemporary literature reporting rates of 20%–40% following cardiac surgery (1, 4). Our ML model achieved AUC values of 0.725 (training), 0.711 (internal validation), and 0.740 (temporal validation), demonstrating moderate and consistent discrimination across different patient cohorts and time periods. The minimal performance degradation between internal and temporal validation (*Δ*AUC = 0.029) supports the stability of the model within our institution; however, this temporal validation does not demonstrate transportability to other hospitals, healthcare systems, or populations, which remains to be established. Furthermore, our findings, derived from the SHAP analysis of the final RF model, indicate that age and operation duration were among the strongest predictors retained by the model.

Feature selection using LASSO regression identified 11 optimal predictors from 56 candidate variables, achieving model parsimony while preserving predictive accuracy. The selected features encompass demographic (age), intraoperative (operation duration), respiratory (SpO2, PaO2, SaO2), metabolic (Lac, ABG K+), hematological (MCV), coagulation (FIB), and hepatic (ALT, ChE) parameters. This multidimensional approach reflects the complex, multifactorial nature of POAF pathophysiology and highlights the importance of integrating diverse physiological data for accurate risk stratification.

Among the identified predictors, ChE emerged as a particularly noteworthy finding. SHAP analysis identified ChE as the most important feature for POAF prediction, exhibiting a robust inverse association with model-predicted AF risk. This finding is consistent with emerging evidence linking cholinergic system dysfunction to cardiovascular pathology (16, 17). ChE, specifically butyrylcholinesterase, an enzyme responsible for hydrolyzing acetylcholine, serves as a surrogate marker of hepatic synthetic function and nutritional status, but may also reflect systemic inflammatory and oxidative stress states (18). Previous studies have demonstrated associations between reduced ChE activity and adverse outcomes in critically ill patients, including prolonged mechanical ventilation, ICU length of stay, and mortality (19). The identification of ChE as a key predictor of POAF in our study is hypothesis-generating. On one hand, it may reflect cholinergic signalling and inflammatory responses relevant to atrial arrhythmogenesis; on the other hand, reduced postoperative ChE may equally indicate impaired hepatic synthetic function, nutritional depletion, haemodilution, systemic inflammation, or greater procedural complexity. Our observational data cannot distinguish among these possibilities and do not establish any causal or mechanistic role of ChE in atrial arrhythmogenesis; analyses adjusting for hepatic function, nutritional, and inflammatory parameters will be important in future prospective studies. The autonomic nervous system plays a crucial role in AF initiation and maintenance, and alterations in cholinergic tone may contribute to atrial electrophysiological remodeling (20). Furthermore, ChE may serve as an integrative marker of hepatic function, nutritional reserve, and systemic stress response, all of which influence postoperative recovery and arrhythmia susceptibility (21).

RF demonstrated the most consistent discrimination compared to alternative algorithms, including LR, DT, SVM, XGBoost, and NB, although its advantage over LR using the same predictors was modest, and RF should therefore be regarded as the most stable—rather than definitively superior—approach among those evaluated. This finding aligns with previous reports highlighting the robustness of ensemble methods for clinical prediction tasks (14). RF's ability to handle nonlinear relationships, accommodate mixed data types, and provide built-in feature importance metrics makes it particularly well-suited for medical prediction modeling (12, 13). The tight concordance between training and validation performance following hyperparameter optimization (*Δ*AUC = 0.014) suggests that overfitting was limited. The performance of our model compares favorably with existing POAF prediction tools. Traditional risk scores such as the HATCH score and CHA₂DS₂-VASc have demonstrated AUC values ranging around 0.70 in cardiac surgery populations (10, 11). Our AUC values (0.71–0.74) fall within the range reported for previously published machine learning models for POAF prediction (14) and should be interpreted as comparable to, rather than exceeding, existing approaches.

The application of SHAP analysis represents a significant strength of this study, addressing the interpretability challenges that often limit clinical adoption of ML models. SHAP values provide both global feature importance rankings and individualized explanations for model predictions, enabling clinicians to understand the contribution of each variable to a patient's predicted risk. This transparency is essential for building trust in ML-based decision support systems and facilitating informed clinical decision-making (22). The identification of nonlinear risk patterns through SHAP dependency plots offers hypothesis-generating insights. The L-shaped relationship between ChE and POAF risk, with an inflection point at approximately 4,000 U/L, suggests an exploratory inflection region below which model-predicted risk increases substantially. Similarly, the accelerated risk gradient for age beyond 70 years and the J-shaped pattern for lactate above 5 mmol/L represent exploratory, model-derived patterns that require formal breakpoint estimation and external validation before any application as clinical cut-off values.

The identified predictors provide insights into the multifactorial pathophysiology of POAF. Advanced age reflects age-related atrial structural and electrical remodeling, including fibrosis, conduction slowing, and calcium handling abnormalities. Prolonged operation duration captures the cumulative effects of surgical trauma, cardiopulmonary bypass exposure, and myocardial ischemia-reperfusion injury, all of which contribute to atrial inflammation and oxidative stress. The inclusion of respiratory parameters (SpO2, PaO2, SaO2) highlights the importance of oxygenation status in POAF development. Hypoxemia promotes sympathetic activation, increases heart rate, and exacerbates oxidative stress, creating a proarrhythmic environment (23). Elevated Lac levels reflect tissue hypoperfusion, anaerobic metabolism, and systemic stress, all of which contribute to atrial myocardial dysfunction and electrical instability (24). Electrolyte abnormalities, particularly potassium derangements, directly affect myocardial repolarization and conduction properties, predisposing to arrhythmia initiation (25). The hematological and coagulation parameters (MCV, FIB) may reflect underlying inflammatory and prothrombotic states that characterize the perioperative period. Hepatic enzymes (ALT, ChE) provide insights into organ function and metabolic reserve, with hepatic dysfunction potentially contributing to drug metabolism abnormalities, coagulation disturbances, and inflammatory dysregulation (26).

The developed prediction framework offers several potential clinical applications. First, early identification of high-risk patients upon ICU admission could help inform decisions regarding prophylactic interventions, including beta-blocker therapy, amiodarone prophylaxis, or magnesium supplementation (27). Second, resource allocation can be optimized by prioritizing high-risk patients for continuous cardiac monitoring and early mobilization protocols. Third, the model facilitates informed patient counseling regarding individual POAF risk and expected postoperative course. Fourth, the identification of modifiable risk factors (e.g., electrolyte optimization, oxygenation management) provides targets for perioperative quality improvement initiatives. The inclusion of immediate postoperative parameters, available within one hour of ICU admission, enables practical risk stratification at a time point when prophylactic interventions may still be effective. This approach balances predictive accuracy with clinical feasibility. It should be emphasized, however, that whether model-guided prophylactic strategies improve patient outcomes remains unknown and requires prospective interventional validation. We emphasize that the present work establishes an interpretable prediction framework and generates hypotheses in a specific single-center, on-pump surgical population, rather than delivering a comprehensive, deployable prediction model.

Several limitations of this study should be acknowledged. First, the retrospective single-center design and exclusive Chinese patient population may limit generalizability, and our validation was temporal rather than geographic; transportability to other hospitals, healthcare systems, and populations requires dedicated external validation. Second, the exclusion of patients with ICU stays shorter than 24 h may have preferentially excluded lower-risk patients with uncomplicated recovery, introducing selection bias that could affect both the observed POAF incidence and model performance. Third, several established POAF predictors were not consistently available in the structured electronic health record fields used for this retrospective extraction—including cardiopulmonary bypass and aortic cross-clamp times, left atrial size, left ventricular ejection fraction, comorbidities such as hypertension, chronic kidney disease and chronic obstructive pulmonary disease, and perioperative prophylactic medication (beta-blockers and antiarrhythmic agents); surgical procedure type was recorded and has now been added to the baseline tables (Table 1 and Supplementary Tables S1, S2), although it was not included among the candidate predictor variables; consequently, our predictor set should be regarded as routinely available rather than comprehensive, operation duration is only a partial surrogate for cardiopulmonary bypass-related inflammation and ischemia-reperfusion injury, and residual confounding is likely. Fourth, although calibration is now reported, the calibration slope above 1 in the temporal validation cohort indicates under-dispersed risk predictions in that cohort, and decision curve analysis and geographic external validation were not performed; these will be integral to the planned prospective multicenter validation, which will also evaluate whether the incremental complexity of random forest is justified over simpler regression approaches and whether immediate postoperative variables add value beyond preoperative and intraoperative information alone. In addition, LASSO feature selection was performed once on the complete training cohort, which does not propagate feature-selection uncertainty into the reported performance estimates. Fifth, the model incorporates only immediate postoperative parameters without capturing dynamic physiological changes during the first 24–48 h. Sixth, the observational design precludes causal inference regarding predictor–POAF relationships, and SHAP-derived patterns are exploratory model-derived descriptions rather than validated clinical thresholds or mechanistic evidence. Finally, prospective multicenter validation is warranted to confirm clinical utility before widespread implementation.

## Conclusion

In summary, this study developed and temporally validated an interpretable RF model for predicting incident POAF following on-pump cardiac surgery. By utilizing LASSO regression to identify 11 optimal predictors and SHAP analysis to reveal nonlinear risk patterns, we identified ChE and age as the predominant model-derived drivers of POAF. The model demonstrated acceptable discrimination and stable performance across internal and temporal validation cohorts, suggesting its potential for early risk stratification in the ICU, although prospective multicenter validation—including prospective evaluation of calibration transportability and model-guided interventions—is required before clinical implementation. The present work should be regarded as an interpretable prediction framework developed in a specific single-center surgical population, generating testable hypotheses—most notably regarding cholinesterase—rather than a comprehensive, ready-to-deploy prediction model.

## Data Availability

The raw data supporting the conclusions of this article will be made available by the authors, without undue reservation.

## Supplementary material

Supplementary Figure S1 Univariate predictive performance of candidate variables. (A) ROC curves for all 56 variables. (B) AUC values ranked by discriminative ability.

Supplementary Figure S2 Performance of the optimized Random Forest model. (A) ROC curve in the training cohort (AUC = 0.725). (B) ROC curve in the internal validation cohort (AUC = 0.711). Shaded areas represent 95% confidence intervals.

Supplementary Figure S3 Exploratory t-SNE visualization of patient-level SHAP vectors in the development cohort (perplexity 5, random seed 2025), with points colored by observed POAF status. The plot is descriptive only and does not constitute independent validation of model discrimination.

## References

[1] Caldonazo T Kirov H Rahouma M Robinson NB Demetres M Gaudino M. Atrial fibrillation after cardiac surgery: a systematic review and meta-analysis. J Thorac Cardiovasc Surg. (2023) 165(1):94–103.e24. 10.1016/j.jtcvs.2021.03.077 33952399

[2] Benedetto U Gaudino MF Dimagli A Gerry S Gray A Lees B. Postoperative atrial fibrillation and long-term risk of stroke after isolated coronary artery bypass graft surgery. Circulation. (2020) 142(14):1320–9. 10.1161/CIRCULATIONAHA.120.046940 33017213 PMC7845484

[3] Son YJ Choi HJ Shim J. Association between postoperative atrial fibrillation after coronary artery bypass grafting and short-term clinical outcomes. BMC Cardiovasc Disord. (2024) 24(1):578. 10.1186/s12872-024-04247-6 39425061 PMC11487808

[4] Seo EJ Hong J Lee HJ Son YJ. Perioperative risk factors for new-onset postoperative atrial fibrillation after coronary artery bypass grafting: a systematic review. BMC Cardiovasc Disord. (2021) 21(1):418. 10.1186/s12872-021-02224-x 34479482 PMC8414730

[5] Maesen B Nijs J Maessen J Allessie M Schotten U. Post-operative atrial fibrillation: a maze of mechanisms. Europace. (2012) 14(2):159–74. 10.1093/europace/eur208 21821851 PMC3262403

[6] Xu H Xu X Ma J Zheng S Song W Zhong Z. Preoperative risk factors for acute postoperative atrial fibrillation in patients undergoing mitral valve repair for degenerative mitral regurgitation: insights into cardiac geometry. Rev Cardiovasc Med. (2025) 26(8):38938. 10.31083/RCM38938 40927107 PMC12415746

[7] Alexandrou K Middleton N Kyranou M Sarafis P. Postoperative atrial fibrillation after coronary artery bypass grafting-clinical, demographic, and intraoperative predictors: a multicenter observational study. Healthcare. (2026) 14(5):690. 10.3390/healthcare14050690 41827643 PMC12985182

[8] Attia A Muthukumarasamy KM Al-U'Datt DGF Hiram R. Relevance of targeting oxidative stress, inflammatory, and pro-resolution mechanisms in the prevention and management of postoperative atrial fibrillation. Antioxidants. (2025) 14(4):414. 10.3390/antiox14040414 40298654 PMC12023940

[9] Zakkar M Ascione R James AF Angelini GD Suleiman MS Suleiman MS. Inflammation, oxidative stress and postoperative atrial fibrillation in cardiac surgery. Pharmacology Therapeutics. (2015) 154:13–20. 10.1016/j.pharmthera.2015.06.009 26116810

[10] Burgos LM Ramírez AG Seoane L Furmento JF Costabel JP Diez M. New combined risk score to predict atrial fibrillation after cardiac surgery: cOM-AF. Ann Card Anaesth. (2021) 24(4):458–63. 10.4103/aca.ACA_34_20 34747754 PMC8617386

[11] Joglar JA Chung MK Armbruster AL Benjamin EJ Chyou JY Cronin EM. 2023 ACC/AHA/ACCP/HRS guideline for the diagnosis and management of atrial fibrillation: a report of the American College of Cardiology/American Heart Association joint committee on clinical practice guidelines. Circulation. (2024) 149(1):e1–e156. 10.1161/CIR.0000000000001193 38033089 PMC11095842

[12] Liu H Zhang W Zhang Y Adegboro AA Fasoranti DO Dai L. Mime: a flexible machine-learning framework to construct and visualize models for clinical characteristics prediction and feature selection. Comput Struct Biotechnol J. (2024) 23:2798–810. 10.1016/j.csbj.2024.06.035 39055398 PMC11269309

[13] Feng J Gong Z Yang J Mo Y Song F. Machine learning-based integration reveals reliable biomarkers and potential mechanisms of NASH progression to fibrosis. Sci Rep. (2025) 15(1):12411. 10.1038/s41598-025-97670-4 40217090 PMC11992153

[14] Guan C Gong A Zhao Y Yin C Geng L Liu L. Interpretable machine learning model for new-onset atrial fibrillation prediction in critically ill patients: a multi-center study. Critical Care. (2024) 28(1):349. 10.1186/s13054-024-05138-0 39473013 PMC11523862

[15] Verma A Sanaiha Y Hadaya J Maltagliati AJ Tran Z Ramezani R. Parsimonious machine learning models to predict resource use in cardiac surgery across a statewide collaborative. JTCVS open. (2022) 11:214–28. 10.1016/j.xjon.2022.04.017 36172420 PMC9510828

[16] Zajonz TS Kunzemann C Schreiner AL Beckert F Schneck E Boening A. Potentials of acetylcholinesterase and butyrylcholinesterase alterations in on-pump coronary artery bypass surgery in postoperative delirium: an observational trial. J Clin Med. (2023) 12(16):5245. 10.3390/jcm12165245 37629287 PMC10455192

[17] Pfenniger A Yoo S Arora R. Oxidative stress and atrial fibrillation. J Mol Cell Cardiol. (2024) 196:141–51. 10.1016/j.yjmcc.2024.09.011 39307416 PMC12266930

[18] Santarpia L Grandone I Contaldo F Pasanisi F. Butyrylcholinesterase as a prognostic marker: a review of the literature. J Cachexia Sarcopenia Muscle. (2013) 4(1):31–9. 10.1007/s13539-012-0083-5 22956442 PMC3581611

[19] Goliasch G Haschemi A Marculescu R Endler G Maurer G Wagner O. Butyrylcholinesterase activity predicts long-term survival in patients with coronary artery disease. Clin Chem. (2012) 58(6):1055–8. 10.1373/clinchem.2011.175984 22294734

[20] Coumel P. Autonomic influences in atrial tachyarrhythmias. J Cardiovasc Electrophysiol. (1996) 7(10):999–1007. 10.1111/j.1540-8167.1996.tb00474.x 8894942

[21] Hajimohammadi S Lockridge O Masson P. New views on physiological functions and regulation of butyrylcholinesterase and potential therapeutic interventions. Front Mol Biosci. (2025) 12:1625318. 10.3389/fmolb.2025.1625318 40612057 PMC12221946

[22] Holzinger A Langs G Denk H Zatloukal K Müller H. Causability and explainability of artificial intelligence in medicine. Wiley Interdiscip Rev Data Min Knowl Discov. (2019) 9(4):e1312. 10.1002/widm.1312 32089788 PMC7017860

[23] May AM Van Wagoner DR Mehra R. OSA and cardiac arrhythmogenesis: mechanistic insights. Chest. (2017) 151(1):225–41. 10.1016/j.chest.2016.09.014 27693594 PMC5989643

[24] Naik R George G Karuppiah S Philip MA. Hyperlactatemia in patients undergoing adult cardiac surgery under cardiopulmonary bypass: causative factors and its effect on surgical outcome. Ann Card Anaesth. (2016) 19(4):668–75. 10.4103/0971-9784.191579 27716698 PMC5070327

[25] Wahr JA Parks R Boisvert D Comunale M Fabian J Ramsay J. Preoperative serum potassium levels and perioperative outcomes in cardiac surgery patients. Multicenter study of perioperative ischemia research group. JAMA. (1999) 281(23):2203–10. 10.1001/jama.281.23.2203 10376573

[26] Chacon MM Schulte TE. Liver dysfunction in cardiac surgery—what causes it and is there anything we can do? J Cardiothorac Vasc Anesth. (2018) 32(4):1719–21. 10.1053/j.jvca.2018.02.037 29573949

[27] Imazio M Brucato A Ferrazzi P Rovere ME Gandino A Cemin R. Colchicine reduces postoperative atrial fibrillation: results of the colchicine for the prevention of the postpericardiotomy syndrome (COPPS) atrial fibrillation substudy. Circulation. (2011) 124(21):2290–5. 10.1161/CIRCULATIONAHA.111.026153 22090167

