# Supplementary material for: Development and validation of an interpretable machine learning model for early prediction of postoperative atrial fibrillation following on-pump cardiac surgery
Source: Front Surg. 2026 Sep 10;13:1933263. doi: 10.3389/fsurg.2026.1933263 (PMC13601210; doi:10.3389/fsurg.2026.1933263)
Supplement: Supplementary file 1 [file Table1.docx]

**TableS1** General characteristics of patients in training and internal validation cohort.

|  | **Training Cohort** | | | | **Internal Validation Cohort** | | | |
| --- | --- | --- | --- | --- | --- | --- | --- | --- |
| **Variable** | **Total**  **(n=609)** | **Non-POAF**  **(n=452)** | **POAF**  **(n=157)** | **p-Value** | **Total**  **(n=261)** | **Non-POAF**  **(n=182)** | **POAF**  **(n=79)** | **p-Value** |
| **Age (yr)** | 61.75 (11.21) | 60.41 (11.44) | 65.59 (9.58) | **<0.001*** | 61.33 (11.80) | 60.61 (11.40) | 62.99 (12.59) | 0.135 |
| **Gender (%)** |  |  |  | 0.921 |  |  |  | 0.779 |
| **Female** | 260 (42.7) | 194 (42.9) | 66 (42.0) |  | 104 (39.8) | 71 (39.0) | 33 (41.8) |  |
| **Male** | 349 (57.3) | 258 (57.1) | 91 (58.0) |  | 157 (60.2) | 111 (61.0) | 46 (58.2) |  |
| **Height (m)** | 1.65 (0.08) | 1.65 (0.08) | 1.64 (0.08) | 0.331 | 1.65 (0.09) | 1.65 (0.09) | 1.66 (0.08) | 0.595 |
| **Weight (kg)** | 66.08 (12.29) | 66.39 (12.23) | 65.19 (12.45) | 0.291 | 67.16 (12.00) | 67.10 (12.28) | 67.28 (11.42) | 0.911 |
| **BMI (kg/m^2^)** | 24.18 (3.61) | 24.24 (3.59) | 24.00 (3.65) | 0.477 | 24.53 (3.50) | 24.56 (3.60) | 24.47 (3.29) | 0.841 |
| **Operation type (%)** |  |  |  | 0.140 |  |  |  | 0.345 |
| **CABG** | 35 (5.7) | 30 (6.6) | 5 (3.2) |  | 11 (4.2) | 6 (3.3) | 5 (6.3) |  |
| **Valve surgery** | 373 (61.2) | 273 (60.4) | 100 (63.7) |  | 165 (63.2) | 111 (61.0) | 54 (68.4) |  |
| **Cardiac tumor resection** | 21 (3.4) | 18 (4.0) | 3 (1.9) |  | 8 (3.1) | 7 (3.8) | 1 (1.3) |  |
| **LVAD implantation** | 14 (2.3) | 7 (1.5) | 7 (4.5) |  | 2 (0.8) | 1 (0.5) | 1 (1.3) |  |
| **Atrial or ventricular septal defect repair** | 22 (3.6) | 18 (4.0) | 4 (2.5) |  | 7 (2.7) | 7 (3.8) | 0 (0.0) |  |
| **Total aortic arch replacement** | 2 (0.3) | 1 (0.2) | 1 (0.6) |  | 4 (1.5) | 3 (1.6) | 1 (1.3) |  |
| **Combined procedures** | 142 (23.3) | 105 (23.2) | 37 (23.6) |  | 64 (24.5) | 47 (25.8) | 17 (21.5) |  |
| **Operation Duration (min)** | 293.00 (95.66) | 284.60 (91.78) | 317.19 (102.56) | **<0.001*** | 298.31 (101.20) | 286.51 (101.66) | 325.48 (95.28) | **0.004*** |
| **HR (bpm)** | 93.09 (15.58) | 92.74 (14.61) | 94.11 (18.10) | 0.343 | 93.57 (15.81) | 93.68 (16.08) | 93.33 (15.29) | 0.871 |
| **RR** | 14.84 (2.18) | 14.86 (2.09) | 14.76 (2.42) | 0.603 | 14.85 (2.00) | 14.72 (1.88) | 15.16 (2.24) | 0.099 |
| **SBP (mmHg)** | 122.33 (19.38) | 123.00 (19.74) | 120.40 (18.24) | 0.148 | 122.29 (18.39) | 122.48 (17.88) | 121.83 (19.62) | 0.793 |
| **DBP (mmHg)** | 65.56 (11.31) | 65.87 (11.48) | 64.68 (10.79) | 0.255 | 65.44 (11.73) | 65.24 (11.50) | 65.92 (12.29) | 0.667 |
| **MBP (mmHg)** | 84.49 (12.59) | 84.91 (12.97) | 83.25 (11.40) | 0.154 | 84.39 (12.46) | 84.32 (12.06) | 84.56 (13.39) | 0.888 |
| **SpO2 (%)** | 99.14 (1.75) | 99.27 (1.51) | 98.75 (2.27) | **0.001*** | 98.98 (1.79) | 99.22 (1.37) | 98.43 (2.42) | **0.001*** |

Data are expressed as mean (SD) for continuous variables and percentage (%) for categorical variables. P value was tested by Student's t-test for continuous variables was tested and Pearson chi-square test for categorical variables. *, P<0.05.

**TableS2** General characteristics and vital signs of patients in temporal validation cohort.

| **Variable** | **Total**  **(n=184)** | **Non-POAF**  **(n=145)** | **POAF**  **(n=39)** | **p-Value** |
| --- | --- | --- | --- | --- |
| **Age (yr)** | 60.13 (11.70) | 58.28 (11.75) | 67.03 (8.60) | **<0.001*** |
| **Gender (%)** |  |  |  | 0.149 |
| **Female** | 69 (37.5) | 50 (34.5) | 19 (48.7) |  |
| **Male** | 115 (62.5) | 95 (65.5) | 20 (51.3) |  |
| **Height (m)** | 1.66 (0.08) | 1.66 (0.08) | 1.63 (0.08) | **0.032*** |
| **Weight (kg)** | 66.77 (12.15) | 67.33 (12.19) | 64.70 (11.95) | 0.232 |
| **BMI (kg/m^2^)** | 24.25 (3.69) | 24.26 (3.75) | 24.22 (3.52) | 0.954 |
| **Operation type (%)** |  |  |  | 0.641 |
| **CABG** | 11 (6.0) | 8 (5.5) | 3 (7.7) |  |
| **Valve surgery** | 121 (65.8) | 94 (64.8) | 27 (69.2) |  |
| **Cardiac tumor resection** | 3 (1.6) | 3 (2.1) | 0 (0.0) |  |
| **LVAD implantation** | 0 (0.0) | 0 (0.0) | 0 (0.0) |  |
| **Atrial or ventricular septal defect repair** | 4 (2.2) | 4 (2.8) | 0 (0.0) |  |
| **Total aortic arch replacement** | 2 (1.1) | 1 (0.7) | 1 (2.6) |  |
| **Combined procedures** | 43 (23.4) | 35 (24.1) | 8 (20.5) |  |
| **Operation Duration (min)** | 293.16 (86.01) | 288.58 (87.47) | 310.21 (79.08) | 0.164 |
| **HR (bpm)** | 90.21 (12.50) | 89.80 (11.49) | 91.72 (15.79) | 0.397 |
| **RR** | 14.64 (1.95) | 14.70 (1.95) | 14.41 (1.92) | 0.405 |
| **SBP (mmHg)** | 124.50 (20.02) | 125.53 (19.76) | 120.70 (20.77) | 0.182 |
| **DBP (mmHg)** | 66.26 (12.05) | 66.50 (12.18) | 65.38 (11.67) | 0.610 |
| **MBP (mmHg)** | 85.68 (13.40) | 86.17 (13.41) | 83.82 (13.40) | 0.332 |
| **SpO2 (%)** | 99.08 (1.94) | 99.09 (2.02) | 99.03 (1.63) | 0.856 |

Data are expressed as mean (SD) for continuous variables and percentage (%) for categorical variables. P value was tested by Student's t-test for continuous variables was tested and Pearson chi-square test for categorical variables. *, P<0.05.

**TableS3** Results of ABG, blood counts, coagulation function test and serum biochemical parameters of patients in temporal validation cohort.

| **Variable** | **Total**  **(n=184)** | **Non-POAF**  **(n=145)** | **POAF**  **(n=39)** | **p-Value** |
| --- | --- | --- | --- | --- |
| **pH** | 7.40 (0.08) | 7.40 (0.07) | 7.39 (0.08) | 0.438 |
| **PaCO2 (mmHg)** | 38.47 (6.84) | 38.40 (6.94) | 38.74 (6.53) | 0.781 |
| **PaO2 (mmHg)** | 173.41 (72.05) | 176.01 (72.55) | 163.72 (70.23) | 0.346 |
| **SaO2 (%)** | 98.83 (1.94) | 98.92 (1.62) | 98.49 (2.83) | 0.212 |
| **BE (mmol)** | -1.00 (3.56) | -0.91 (3.46) | -1.35 (3.95) | 0.496 |
| **PaO2/FiO2** | 303.29 (130.20) | 309.58 (135.51) | 279.90 (106.50) | 0.207 |
| **ABG-Na (mmol)** | 139.92 (3.94) | 140.03 (3.81) | 139.51 (4.39) | 0.470 |
| **ABG-K (mmol)** | 4.07 (0.58) | 4.08 (0.57) | 4.04 (0.61) | 0.727 |
| **ABG-Ca (mmol)** | 1.11 (0.08) | 1.11 (0.08) | 1.11 (0.08) | 0.930 |
| **Lac (mmol)** | 4.24 (3.00) | 4.22 (3.06) | 4.29 (2.80) | 0.900 |
| **WBC (10^9^/L)** | 12.78 (4.96) | 12.64 (5.04) | 13.28 (4.66) | 0.474 |
| **Lymphocyte (10^9^/L)** | 0.85 (0.52) | 0.84 (0.48) | 0.88 (0.66) | 0.630 |
| **Monocyte (10^9^/L)** | 0.58 (0.40) | 0.57 (0.39) | 0.62 (0.43) | 0.532 |
| **Neutrophil (10^9^/L)** | 11.31 (4.54) | 11.19 (4.66) | 11.74 (4.07) | 0.499 |
| **Eosinophil (10^9^/L)** | 0.03 (0.04) | 0.03 (0.05) | 0.03 (0.02) | 0.669 |
| **Basophil (10^9^/L)** | 0.01 (0.01) | 0.01 (0.01) | 0.01 (0.01) | 0.741 |
| **RBC (10^12^/L)** | 3.58 (0.53) | 3.59 (0.54) | 3.55 (0.49) | 0.703 |
| **Hb (g/L)** | 108.12 (16.15) | 108.23 (16.32) | 107.72 (15.71) | 0.862 |
| **HCT (%)** | 32.75 (4.66) | 32.76 (4.66) | 32.69 (4.72) | 0.939 |
| **MCV (fL)** | 91.69 (4.91) | 91.56 (4.73) | 92.17 (5.56) | 0.487 |
| **MCH (pg)** | 30.25 (1.78) | 30.22 (1.69) | 30.38 (2.10) | 0.608 |
| **MCHC (g/L)** | 329.92 (8.98) | 330.03 (8.96) | 329.51 (9.18) | 0.752 |
| **RDW (%)** | 13.23 (1.22) | 13.23 (1.28) | 13.25 (0.97) | 0.898 |
| **PLT (10^9^/L)** | 133.56 (39.00) | 134.31 (39.77) | 130.77 (36.34) | 0.616 |
| **PCT (%)** | 0.14 (0.04) | 0.14 (0.04) | 0.13 (0.04) | 0.744 |
| **MPV (fL)** | 10.26 (1.10) | 10.26 (1.15) | 10.27 (0.92) | 0.952 |
| **PDW (%)** | 12.78 (4.96) | 12.64 (5.04) | 13.28 (4.66) | 0.474 |
| **PT (s)** | 16.16 (0.96) | 16.12 (1.06) | 16.33 (0.32) | 0.225 |
| **INR** | 12.98 (1.35) | 13.02 (1.39) | 12.83 (1.16) | 0.426 |
| **APTT (s)** | 1.13 (0.12) | 1.14 (0.13) | 1.12 (0.10) | 0.432 |
| **FIB (g/L)** | 28.92 (6.50) | 29.18 (7.18) | 27.96 (2.58) | 0.297 |
| **TT (s)** | 2.81 (0.82) | 2.78 (0.84) | 2.93 (0.72) | 0.298 |
| **D-dimer(mg/L)** | 25.50 (20.23) | 26.67 (22.28) | 21.15 (8.04) | 0.130 |
| **K (mmol)** | 4.40 (0.57) | 4.42 (0.55) | 4.35 (0.64) | 0.541 |
| **Na (mmol)** | 141.11 (3.99) | 141.15 (3.89) | 140.96 (4.39) | 0.794 |
| **Cl (mmol)** | 103.77 (4.30) | 103.87 (4.18) | 103.39 (4.78) | 0.540 |
| **Ca (mmol)** | 2.35 (0.16) | 2.34 (0.16) | 2.36 (0.15) | 0.497 |
| **ALT (U/L)** | 45.14 (76.86) | 49.10 (85.92) | 30.40 (13.77) | 0.178 |
| **AST (U/L)** | 112.58 (161.15) | 119.26 (178.84) | 87.75 (55.34) | 0.280 |
| **BUN (mmol)** | 9.21 (3.73) | 9.10 (3.70) | 9.62 (3.85) | 0.443 |
| **Cr (umol)** | 88.96 (59.01) | 88.38 (62.94) | 91.13 (41.83) | 0.797 |
| **GLU (mmol)** | 9.80 (2.87) | 9.55 (2.78) | 10.71 (3.05) | **0.025*** |
| **TBIL (umol)** | 34.33 (12.46) | 34.00 (12.53) | 35.53 (12.26) | 0.498 |
| **DBIL (umol)** | 10.64 (5.53) | 10.50 (5.48) | 11.14 (5.76) | 0.523 |
| **IBIL (umol)** | 23.69 (9.41) | 23.50 (9.56) | 24.39 (8.91) | 0.604 |
| **ALP (U/L)** | 49.52 (21.82) | 48.95 (21.43) | 51.64 (23.41) | 0.497 |
| **GGT (U/L)** | 35.47 (39.17) | 34.97 (37.16) | 37.33 (46.36) | 0.739 |
| **ChE (U/L)** | 5485.18 (1021.43) | 5519.90 (1064.14) | 5356.09 (843.51) | 0.375 |
| **UA (umol)** | 326.23 (70.64) | 328.07 (70.80) | 319.42 (70.53) | 0.499 |
| **TP (g/L)** | 64.19 (6.47) | 64.05 (6.15) | 64.73 (7.62) | 0.560 |
| **GLB (g/L)** | 23.11 (3.16) | 23.14 (3.10) | 23.01 (3.45) | 0.822 |
| **ALB (g/L)** | 41.08 (5.34) | 40.91 (5.19) | 41.72 (5.87) | 0.401 |

Data are expressed as mean (SD) and P value was tested by Student's t-test. *, P<0.05.
